# Stratification of patients with Menière’s disease based on eye movement videos recorded from the beginning of vertigo attacks and contrast-enhanced MRI findings

**DOI:** 10.3389/fneur.2023.1348177

**Published:** 2024-01-11

**Authors:** Yuya Ueno, Takao Imai, Kayoko Higashi-Shingai, Yumi Ohta, Takashi Sato, Takefumi Kamakura, Yoshiyuki Ozono, Hidenori Inohara

**Affiliations:** ^1^Department of Otorhinolaryngology-Head and Neck Surgery, Osaka University Graduate School of Medicine, Osaka, Japan; ^2^Department of Otorhinolaryngology and Head and Neck Surgery, Bellland General Hospital, Osaka, Japan

**Keywords:** Menière’s disease, endolymphatic hydrops, nystagmus recording, inner ear, diagnostic criteria, nystagmus direction, contrast-enhanced MRI

## Abstract

**Purpose:**

Diagnosis of Menière’s disease (MD) relies on subjective factors and the patients diagnosed with MD may have heterogeneous pathophysiologies. This study aims to stratify MD patients using two objective data, nystagmus videos and contrast-enhanced magnetic resonance imaging (CE-MRI).

**Methods:**

This is a retrospective cross-sectional study. According to the Japan Society for Equilibrium Research criteria (c-JSER), adults diagnosed with definite MD and who obtained videos recorded by portable nystagmus recorder immediately following vertigo attacks and underwent CE-MRI of the inner ear were included (*ss* = 91). Patients who obtained no nystagmus videos, who had undergone sac surgery, and those with long examination intervals were excluded (*n* = 40).

**Results:**

The gender of the subjects was 22 males and 29 females. The age range was 20–82 y, with a median of 54 y. Endolymphatic hydrops (EH) were observed on CE-MRI in 84% (43 patients). Thirty-one patients had unilateral EH. All of them demonstrated EH on the side of the presence of cochlear symptoms. The number of patients who had both nystagmus and EH was 38. Five patients only showed EH and 5 patients only exhibited nystagmus, while 3 patients did not have either. Of the 43 nystagmus records, 32 showed irritative nystagmus immediately after the vertigo episode. The direction of nystagmus later reversed in 44% of cases over 24 h.

**Conclusion:**

Patients were stratified into subgroups based on the presence or absence of EH and nystagmus. The side with cochlear symptoms was consistent with EH. The c-JSER allows for the diagnosis of early-stage MD patients, and it can be used to treat early MD and preserve hearing; however, this approach may also include patients with different pathologies.

## 1 Introduction

Menière’s disease (MD), one of the most common causes of episodic vertigo (1), causes a variety of ear symptoms, including vertigo, sensorineural hearing loss, tinnitus, and ear fullness (2), and the attacks begin without any signs. MD is the second most common form of inner ear vertigo after benign paroxysmal positional vertigo, with a prevalence of 50 per 100,000 individuals in Japan (3, 4). MD is a major obstacle to the quality of life of patients, especially when patients are in their prime working years; it affects their social and economic activities. Patients with MD reportedly show endolymphatic hydrops (EH) of the inner ear on contrast-enhanced magnetic resonance imaging (CE-MRI) (5). EH is the expansion of the endolymphatic space in the cochlea and the vestibule.

In Japan, the diagnostic criteria for MD published by the Japan Society for Equilibrium Research (JSER) (6) are used clinically because the clinical guideline for MD in Japan targets cases diagnosed based on the criteria of JSER (c-JSER). In the insurance system maintained by the Japanese government, MD is covered for patients diagnosed with MD by c-JSER. The c-JSER basically follows the Bárány Society criteria, and confirmation of EH by autopsy or CE-MRI is not required to diagnose definite MD. The diagnosis for MD is based on a questionnaire, and there are no specific biomarkers, such as those found in malignant tumors, so patients diagnosed with MD may be a heterogeneous population with various pathogenesis. Some patients with MD have EH on CE-MRI, but others do not (7). Many therapies for MD exist, for example, oral administration of diuretics (8), intratympanic administration of steroids (9), intratympanic administration of gentamicin (10), endolymphatic sac decompression surgery (11), and vestibular neurectomy (12). However, there is no evidence for the effectiveness of any of these treatments. This might be because we are applying a uniform treatment to a population with different pathologies.

Regarding the examination, pure tone audiometry is a subjective examination and is not a completely objective test. On the contrary, nystagmus is the definitive objective finding. MD reportedly causes irritative nystagmus (IN; horizontal or horizontal torsional nystagmus beating to the affected side), which reverses in direction over time to paralytic nystagmus (PN; horizontal or horizontal torsional nystagmus beating to the unaffected side) (13). Although IN and PN are typical nystagmus findings in patients with MD, this nystagmus may not be observed clearly in outpatients because they come to the hospital at intervals of episodes of vertigo attacks. The patients sometimes show PN after shaking their heads, but not always.

**Figure 1:**
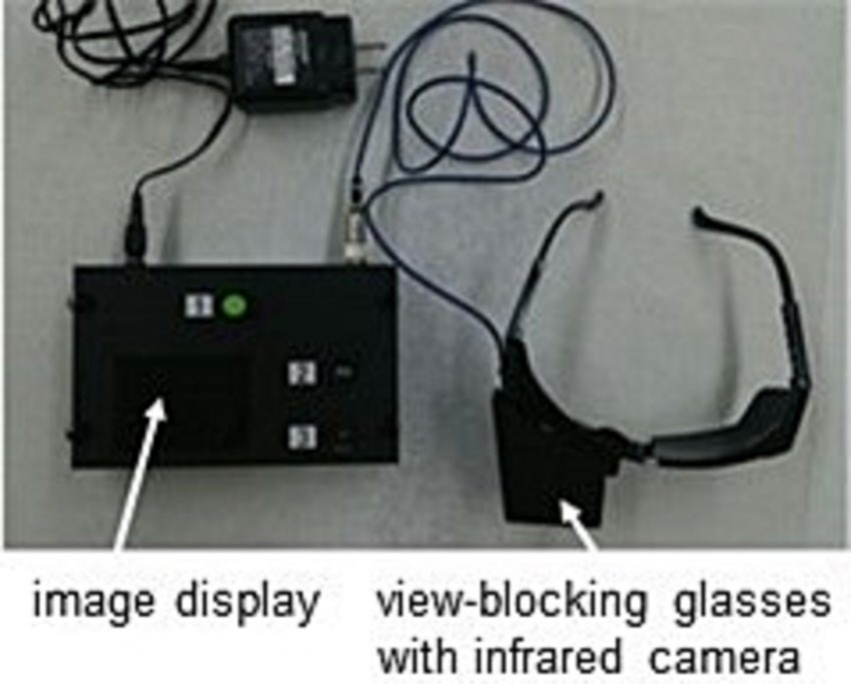
Photo of Portable nystagmus recorder (PNR). A PNR consists of a portable video recorder and an infrared charge-coupled device (CCD) camera. The eyes were covered with glasses, and nystagmus was recorded in the dark. The PNR needed only a simple method in which the patient pressed three buttons in order.

The purpose of this study is to stratify patients with definite MD who have been diagnosed by c-JSER, which relies on questionnaires and subjective pure tone audiometry tests, using purely objective tests, namely nystagmus observations and CE-MRI. Nystagmus observations can only be obtained during vertigo attacks. Therefore, we developed a portable nystagmus recorder (PNR) capable of recording nystagmus during vertigo attacks (Figure 1). Nystagmus can be recorded from the beginning of vertigo attacks with a simple operation performed by the patient (Supplementary Video 1). We used PNR and accumulated video data of nystagmus in patients with MD. In addition, we discussed the pathophysiological aspects within each stratified subgroup.

## 2 Materials and methods

### 2.1 Guidelines and ethics

The study was conducted in accordance with the Declaration of Helsinki. This cross-sectional study was based on Strengthening the Reporting of Observational studies in Epidemiology (STROBE) reporting guidelines. This study was approved by the Ethics Committee of the Osaka University Hospital (No. 22282). Written informed consent was obtained from all the participants.

### 2.2 Study population and participant selection

**Figure 2:**
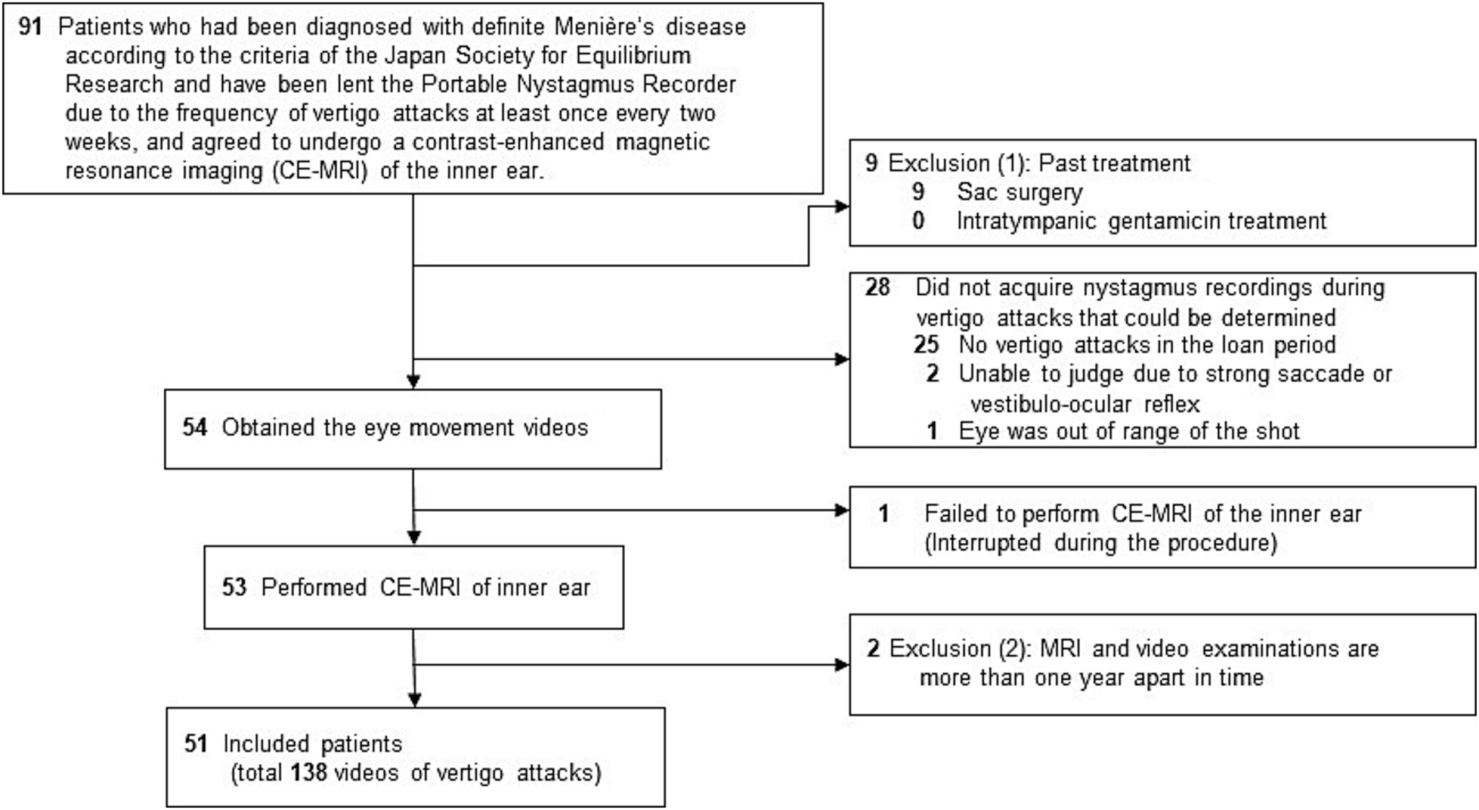
Flowchart of patient inclusion. Patients were required to record vertigo attacks during the observation period using a portable nystagmus recorder (PNR). It was provided to patients who experienced vertigo attacks at least once every two weeks. A history of treatment such as sac surgery or intratympanic gentamicin treatment could affect the characteristics of nystagmus, and such patients were excluded. Patients with a period of >1 y between each examination were excluded since the pathogenesis may have changed during this period.

**Figure 3:**
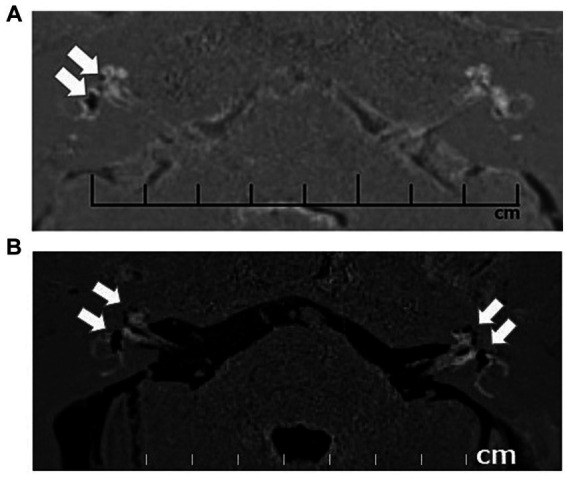
Contrast-enhanced magnetic resonance images (CE-MRI) of unilateral and bilateral endolymphatic hydrops. Unilateral (right) endolymphatic hydrops **(A)** and bilateral endolymphatic hydrops **(B)**. Arrows indicate the endolymphatic hydrops in the cochlea and vestibule.

Data were retrospectively extracted from the electronic medical records database of the Osaka University Hospital and the database of nystagmus videos. Inclusion criteria were (Figure 2): (i) men or women ≥20 y who visited the Department of Otorhinolaryngology-Head and Neck Surgery at the Osaka University Hospital between December 2015 and April 2022, (ii) patients diagnosed with definite MD according to the c-JSER (6), (iii) patients reporting at least one vertigo attack every 2 weeks and those who were provided a PNR, and (iv) those who agreed to undergo CE-MRI of the inner ear (*n* = 91) (Figure 3). Patients were excluded if they met any of the following conditions: (i) had undergone sac surgery (*n* = 9) or intratympanic gentamicin treatment (*n* = 0), (ii) had failed to obtain at least one video of the eye movements from the beginning of the vertigo attacks using the PNR (*n* = 28) or had failed to undergo CE-MRI (*n* = 1), and (iii) had a period of more than 1 year between the first use of the PNR and CE-MRI (n = 2). Consequently, 51 patients (138 videos of vertigo attacks) were included in the study (Table 1).

**Table 1:** Participant characteristics and details of CE-MRI findings.

Characteristics	No. (%)
Sex	
Men	22 (43)
Women	29 (57)
Age	
Range, y	20–82
Median, y	54
Laterality	
Right	18 (35)
Left	33 (65)
Severity(AAO-HNS)	
Stage I	11 (22)
Stage II	3 (6)
Stage III	27 (53)
Stage IV	10 (20)
The time between CE-MRI and the first video capture	
Range, days	1–332
Median, days	48
Details of CE-MRI findings	
Manufacturer of the MRI equipment	
Siemens	16 (31)
GE Healthcare	35 (69)
Presence of endolymphatic hydrops	
No	8 (16)
Yes	43 (84)
Laterality	
Unilateral	31 (72)
Bilateral	12 (28)
Position	
Cochlea, ears	47 (85)
Vestibule, ears	45 (82)

CE-MRI, contrast-enhanced magnetic resonance imaging.

### 2.3 Determination of the affected side

**Figure 4:**
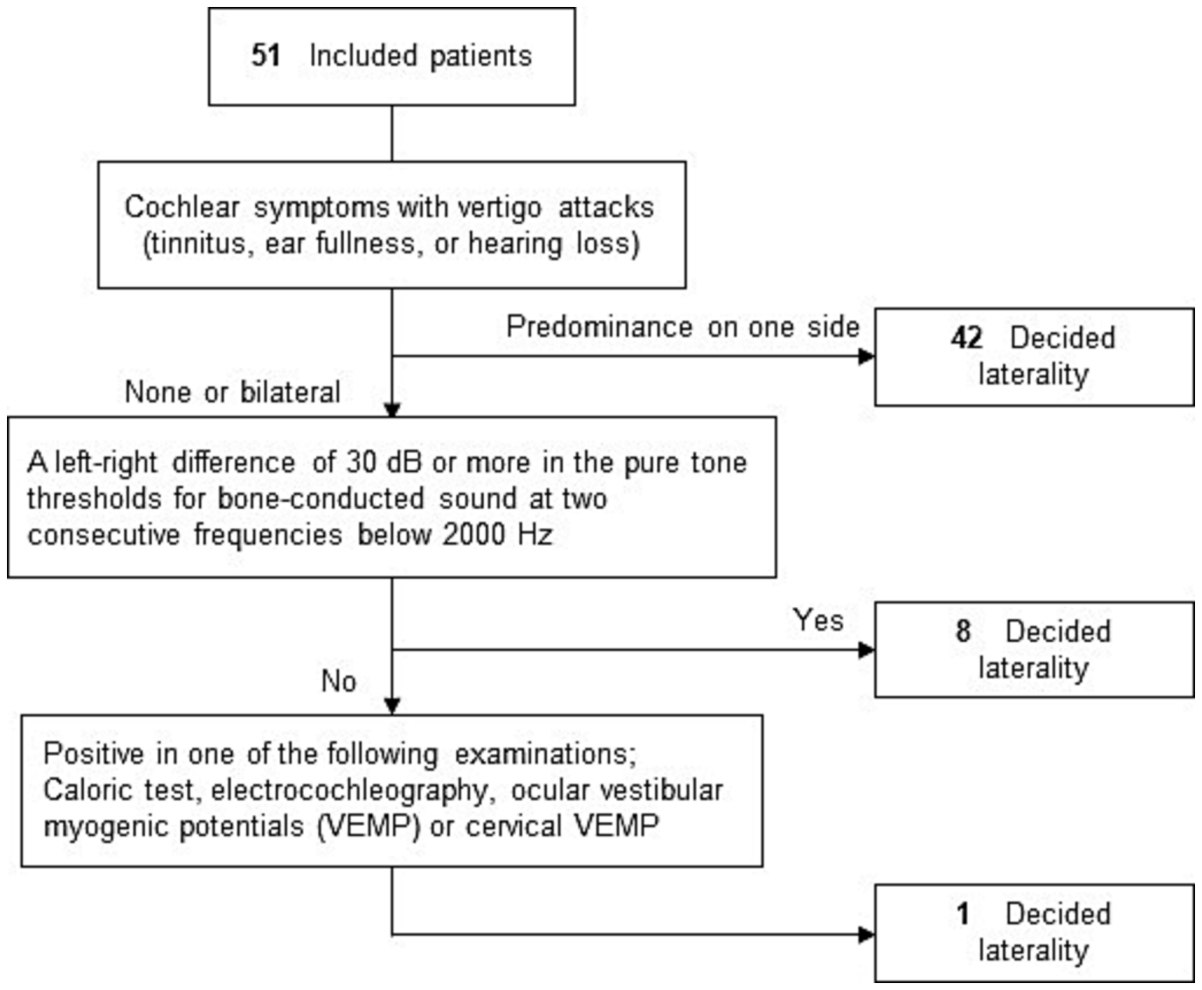
Flowchart of laterality decision. To determine whether the nystagmus is irritative or paralytic, this flowchart sorted out the affected side as unilateral. If the bilateral MD is applied to the flowchart, the affected side will not be decided.

While determining the affected side, we did not initially rely on CE-MRI or the direction of nystagmus. Instead, we first determined the affected side mainly with a questionnaire. We then subsequently assessed the relationship between the affected side, the side presence of EH on CE-MRI, and the direction of nystagmus. The MD-affected side of all the patients was determined using a flowchart based on a questionnaire on cochlear symptoms during vertigo attacks and the results of various clinical examinations (Figure 4) (14–16). The measurement method for electrocochleography and glycerol vestibular evoked myogenic potential (GVEMP) followed previously reported protocols (17, 18). To determine whether the nystagmus was irritative or paralytic, this flowchart determined the affected side as unilateral. If both the right and left ear had cochlear symptoms, hearing fluctuations, and clinical examinations, the patients would be diagnosed as bilateral MD. Even if EH in the bilateral inner ear was observed, the patient was diagnosed as unilateral MD when the patient had unilateral cochlear symptoms with our flowchart. There were no patients diagnosed as bilateral MD according to the flowchart in this study.

### 2.4 Nystagmus recording from the beginning of vertigo attacks

A PNR (Figure 1) was provided to the patients with MD to record nystagmus from the beginning of vertigo attacks. The PNR was manufactured by the Biomedica Corporation (Osaka, Japan) and consisted of a portable video recorder and an infrared charge-coupled device (CCD) camera. The eyes were covered with glasses, and nystagmus was recorded in the dark. The PNR needed only a simple method in which the patient pressed three buttons. The observation period was 2 weeks and was extended for another 2 weeks if no vertigo attacks were recorded. Patients submitted one or more video series according to the number of vertigo attacks they experienced.

The direction of nystagmus recorded in the videos was determined as follows: IN, PN, or undefinable for each video, with the affected side as the reference. Videos without nystagmus were considered undefinable. Only a few vertical nystagmus was also observed but this was classified as undefinable when the horizontal component was unclear. There was no reversal of nystagmus in a single recording video. Typical nystagmus directions for each patient were determined from the recording results when the patients recorded multiple attacks. The results at the onset of the attack and the others were compared. Y. U. and T. I. independently reviewed all the videos and discussed their findings to reach a consensus regarding the type of nystagmus observed.

### 2.5 CE-MRI of the inner ear

All the enrolled patients underwent CE-MRI using one of two types of MRI equipment: MAGNETOM Trio (Siemens Medical Solutions, Erlangen, Germany) (Figure 3) or Sigma Excite HD 3 T (GE Healthcare, UK). The CE-MRI technique and determination of the presence or absence of EH in the cochlea and vestibule were the same as those employed in previous studies (19, 20).

### 2.6 Statistical analysis

We tested the independence of two items: the presence of EH on CE-MRI and the presence of nystagmus during vertigo attacks. We examined the statistical significance of the difference in the presence of nystagmus between EH-positive and EH-negative patients. Statistical analysis was performed using Easy R (EZR) software (Jichi Medical University Saitama Medical Center, Saitama, Japan); 2 × 2 tables were generated, and Fisher’s exact test was applied. Statistical significance was set at *p* < 0.05.

## 3 Results

### 3.1 Patients

The background characteristics of the included patients are shown in Table 1. Twenty-two (43%) patients were men, and 29 (57%) were women; the age range was 20–82 y, with a median age of 54 y. The affected side, as determined from the flowchart (Figure 4), was the right and left in 18 (35%) and 33 (65%) patients, respectively. According to the severity classification of AAO-HNS, there were 11 patients in Stage I, 3 in Stage II, 27 in Stage III, and 10 in Stage IV. The time between CE-MRI and the first video capture ranged from 1–332 days, with a median of 48 days.

### 3.2 Contents of the video data

**Figure 5:**
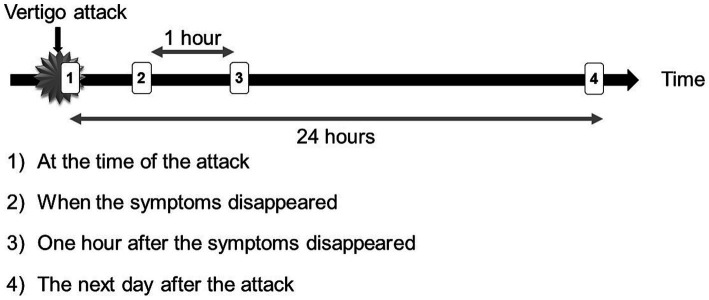
Recording Timing. Patients were instructed to use the PNR from the beginning of vertigo attacks to record videos and each recording lasted 2 min and to record four times per vertigo attack. We have collected video data taken (1) at the time of the attack, (2) when the symptoms disappeared, (3) one hour after the symptoms disappeared, and (4) the day after the attack. Sometimes the patients missed recording videos (2 and 3) due to going out or sleeping.

**Figure 6:**
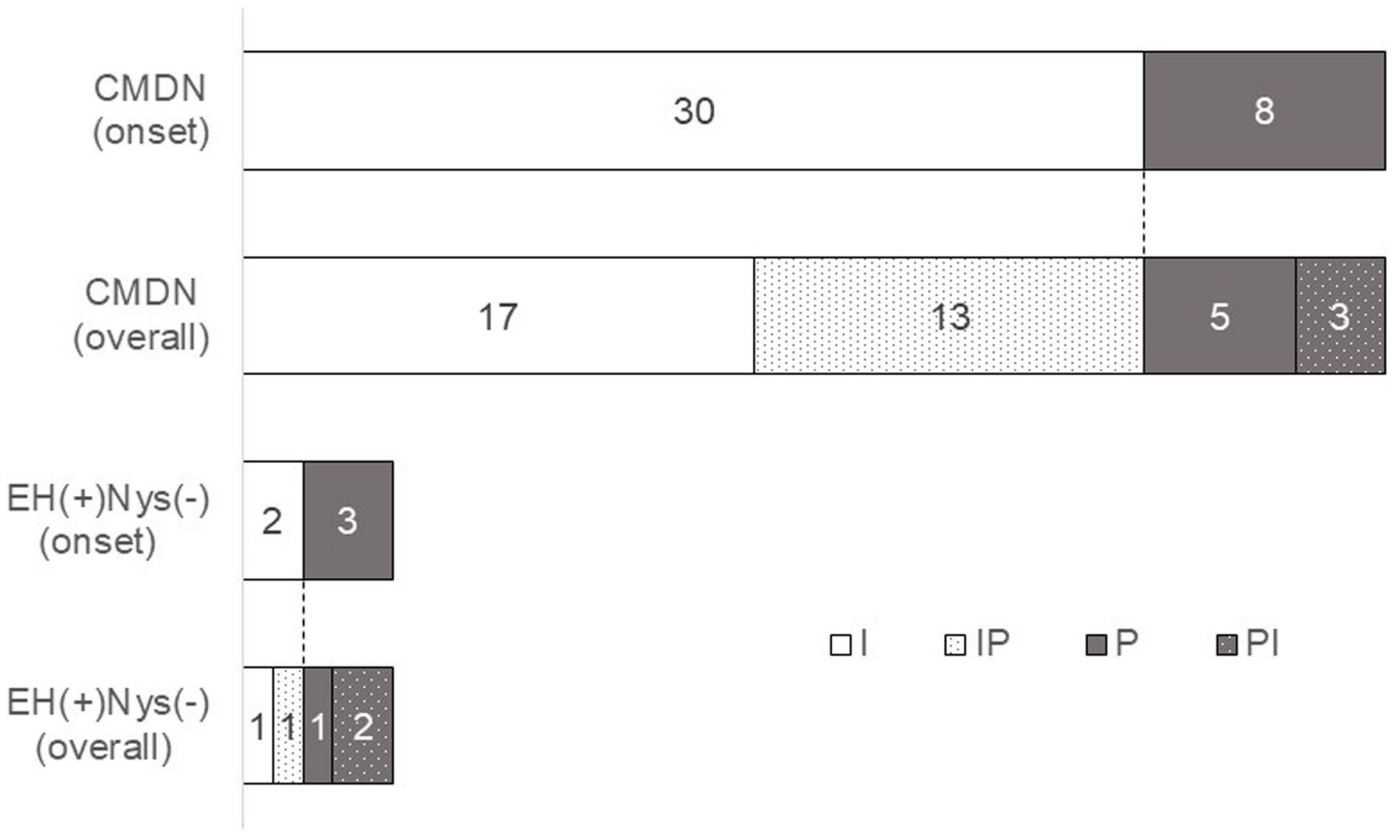
Characteristics of temporal changes in nystagmus direction. We observed the nystagmus of 38 patients in CMDN and 5 patients without EH. We checked all videos and determined the representative patterns of nystagmus in each 43 patients. In CMDN, most of the initial nystagmus were IN (30/38, 79%) and 43%(13/30) of them showed reversals in 24 h. 8 patients in the rest of CMDN showed PN at the onset of the vertigo attack, and 38%(3/8) showed reversals. On the other hand, among the 5 patients without EH, 2 patients showed IN, 3 showed PN, 1 case with IN and 2 cases with PN showing reversal the next day. I, patients with irritative nystagmus. IP, patients with irritative nystagmus followed by paralytic nystagmus, P, patients with paralytic nystagmus, PI, patients with parallytic nystagmus followed by irritative nystagmus.

In total, 138 attacks were recorded from 51 patients. The number of vertigo attacks recorded per patient ranged from one to nine. The median duration of a subjective vertigo attack (the time difference between the second recording moment of vertigo disappearance and the initiation of the first recording) was 2 h and 8 min, and the duration between the first and last recordings of a single vertigo attack was 24 h and 1 min. The direction of nystagmus at the onset of the vertigo attack included 73 (53%) attacks of IN, and 35 (25%) attacks of PN, and the remaining 30 (22%) video recordings were undefinable (Table 2). In the case-by-case analysis, 25 (49%) patients showed only IN, and nine (18%) patients showed only PN. Nine patients (18%) showed both IN and PN with multiple vertigo attacks. Eight patients (15%) had no nystagmus in the video. We recorded three times following a single vertigo attack (at the time the symptom disappeared, one hour after the symptom disappeared, and the day after the attack) (Figure 5). Comparing the direction at the onset of the attack, if the other three recordings showed the reverse direction, we concluded the reversal of nystagmus occurred. The reversal of nystagmus was observed in 19 patients (44%), of these, 14 were from IN to PN, and 5 were from PN to IN (see Figure 6).

**Table 2:** Details of nystagmus in the videos.

Characteristics	No. (%)
**Number of videos by each type of nystagmus**	
IN	73 (53)
PN	35 (25)
Undefinable^a^	30 (22)
**Number of patients by nystagmus direction at the onset of the vertigo attack**	
IN only	25 (49)
PN only	9 (18)
Both IN and PN^b^	9 (18)
Undefinable	8 (15)
**Number of patients whose nystagmus reversed among 43 showing nystagmus**	
IN to PN	14 (33)
PN to IN	5 (12)
total	19 (44)

a Video without nystagmus or pure vertical nystagmus.

b This means that the patients recorded multiple vertigo attacks and video 1) (Figure 5). contained both IN and PN.

IN. irritative nystagmus; PN, paralytic nystagmus.

### 3.3 CE-MRI findings

Of the 51 patients in which CE-MRI was performed, 16 (31%) were performed using Siemens MRI equipment and 35 (69%) with GE Healthcare equipment, as described in the Methods section (Table 1). The overall EH positivity rate in the patients included in this study was 84% (43/51 patients), comparable to previous reports (21, 22). Of the 43 patients, 31 (72%) had unilateral EH and 12 (28%) had bilateral EH, respectively. All 31 patients with unilateral EH demonstrated EH on the affected side, as determined by the flowchart (Figure 4). We also identified whether patients had EH in the cochlea and/or vestibule within the inner ear. Of the 43 patients with EH, the total number of ears with EH was 55. The number of ears with EH in the cochlea was 47 (85%) and that in the vestibule was 45 (82%).

### 3.4 Stratification of the patients using two objective findings: Nystagmus videos during the vertigo attacks and CE-MRI

We stratified all 51 patients into four subgroups based on two objective findings: the presence of nystagmus in videos and the CE-MRI findings in the inner ear (Table 3). The number of patients with nystagmus and EH was 38 (74%), nystagmus only was 5 (10%), EH only was 5 (10%), and neither nystagmus nor EH was 3 (6%), respectively. Statistical analysis revealed no significant difference in the prevalence of nystagmus between EH-positive and EH-negative patients (*p* = 0.10, Table 3).

**Table 3:** Stratification of the patients using nystagmus video recordings and CE-MRI of the inner ear.

	No.		
Nystagmus	EH+	EH-	Total
Yes^a^	38	5	43
No	5	3	8
Total	43	8	51

*p* = 0.10 (Fisher’s exact test).

^a^Direction of nystagmus not considered. EH, endolymphatic hydrops.

We named the subgroup of patients who exhibited nystagmus and EH as a certain MD with nystagmus (CMDN). In this study, 38 out of 51 patients were in the CMDN group. Records of nystagmus were obtained in 38 patients of CMDN and 5 patients with nystagmus-positive and EH-negative, and the direction of nystagmus was investigated (Figure 6). We checked all videos and determined the representative patterns of nystagmus in each 43 patients. In CMDN, most of the initial nystagmus were IN (30/38, 79%) and 43%(13/30) of them showed reversals in 24 h. 8 patients in the rest of CMDN showed PN on the onset of the vertigo attack, and 38%(3/8) showed reversals. On the other hand, among the 5 patients without EH, 2 patients showed IN, and 3 showed PN, with 1 case with IN and 2 cases with PN showing reversal the next day.

Table 4 lists the results of inner ear function tests for 13 cases in which either nystagmus or EH were not observed. The subgroups listed from top to bottom are with nystagmus but without EH, without nystagmus and with EH, and neither nystagmus nor EH. The cases with smaller numbers, i.e., those shown higher, are more characteristic of MD. That is, cases within each subgroup with hearing fluctuations or abnormalities in inner ear function tests are listed higher. In the subgroup with nystagmus but without EH, except for one case, the hearing was almost normal with levels within 25 dB and hearing fluctuations between 10–25 dB. In the subgroup without nystagmus but with EH, except for one case, the hearing was ≤25 dB, but hearing fluctuations ranged widely from 15–55 dB. The subgroup without nystagmus nor EH had normal hearing and inner ear function, with all cases having hearing levels ≤7 dB and hearing fluctuations within 20 dB. The results of the inner ear function tests such as GVEMP and electrocochleography (15, 16, 23) showed positive findings of EH in nearly half of the cases.

**Table 4:** Clinical examination data for each non-CMDN (certain Menière’s disease with nystagmus) patient.

Patient No.	Hearing^a^, dB	Fluctuating range of hearing^b^, dB	GVEMP	electrocochleography
Subgroup with nystagmus but without EH (*n* = 5)				
1	9	25	positive	negative
2	45	20	negative	positive
3	7	15	NT	NT
4	23	15	NT	negative
5	15	10	NT	NT
Subgroup without nystagmus and with EH (*n* = 5)				
6	25	55	NT	positive
7	23	45	negative	negative
8	24	45	NT	NT
9	46	35	NT	positive
10	0	15	NT	negative
Subgroup that had neither nystagmus nor EH (*n* = 3)				
11	7	20	NT	negative
12	1	15	NT	NT
13	6	10	NT	NT

a Average threshold of frequencies ≤ 2000 Hz for best hearing on the affected side.

b Maximum fluctuating range for each frequency ≤ 2000 Hz on the affected side.GVEMP, glycerol vestibular evoked myogenic potentials test; EH, endolymphatic hydrops; NT, not tested.

## 4 Discussion

### 4.1 Considerations for the four subgroups

In this study, we stratified the subgroup of patients who exhibited nystagmus and EH as CMDN (Table 3). In the CMDN group, all patients showed EH on the affected side, and nearly 80% of patients in this group presented with IN at the time of onset of the attack (Figure 6). This means that EH causes IN, i.e., the pathophysiology of the vertigo attack in CMDN is EH in the inner ear, and the pathophysiology of definite MD diagnosed by the c-JSER is EH in the inner ear because CMDN represented most patients in the study (38/51, 74.5%). We included only the patients with frequent vertigo attacks, at least once in two weeks, so according to the severity classification of AAO-HNS, most of the patients were categorized in Stage III and IV (37/51, 73%). They were advanced and their EH was detected clearly.

However, some patients were stratified into another subgroup, suggesting that the population diagnosed with definite MD using the c-JSER had a little heterogeneity. The results did not show statistical significance between the subgroup with nystagmus (88%, 38/45) with more EH than those without nystagmus (63%, 5/8) during vertigo attack (*p* = 0.10, Table 3). The lack of statistically significant differences may be because of the small number of cases in the subgroup without nystagmus.

We presented the clinical examination data for each patient in the subgroup with nystagmus but without EH in Table 4. The fluctuating range of hearing test results, GVEMP, and electrocochleography findings are highly specific for EH (15, 16, 23). Cases with more markedly positive results are shown in the upper row of Table 4. This subgroup may have early-onset EH that is too small to be detected by CE-MRI (24), i.e., the pathophysiology of patients stratified in this subgroup is also EH. In the future, EH may be observed in this patient group with CE-MRI. Since this group has less fluctuation in hearing level, it may be possible to keep their hearing in good condition by providing therapeutic intervention early on before their hearing deteriorates. In particular, if other test results suggest EH than CE-MRI, early intervention should be performed.

Alternatively, there is a possibility that the pathophysiology is something other than EH, for example, vestibular migraine (VM). However, all patients included in this study were excluded from the diagnosis of VM at the time of diagnosis. Therefore, all patients in this subgroup did not fill the diagnostic criteria of VM, such as “current or previous history of migraine with or without aura according to the International Classification of Headache Disorders” and/or “one or more migraine features with at least 50% of the vestibular episodes headache with at least two of the following characteristics: one-sided location, pulsating quality, moderate or severe pain intensity, aggravation by routine physical activity, photophobia and phonophobia, visual aura (25).” However, in the future, they may claim headaches that meet the diagnostic criteria for migraine, or the migraine-associated vertigo attacks may exceed 50%. Careful following up with patients in this group to determine whether such events occur is necessary.

The range of hearing fluctuations was greater than expected in the subgroup with EH but without nystagmus (Table 4, middle panel). Furthermore, many patient complaints and other test results were consistent with MD. Because patients in this group met the diagnostic criteria of definite MD and had EH on the affected side, the idea that their pathophysiology is other than EH is unreasonable. They did not show nystagmus during the vertigo attack because the observation period using the PNR may have been too short for recording nystagmus. Nystagmus could have been captured upon extending the observation period using PNR.

In the subgroup with neither nystagmus nor EH, all patients had an almost normal sense of hearing. Although it might occur that these patients were early MD and their nystagmus was so small and was determined as no nystagmus, the results indicated the inner ear is intact functionally and morphologically. Despite meeting diagnostic criteria for definite MD, three cases consistently lacked nystagmus and EH. These vertigo episodes might have had origins other than MD during the testing period. We consider that it is highly likely that the three patients would be diagnosed with another form of vertigo through blood pressure tests or psychological tests. Because the origin of their vertigo attack might not be the inner ear, the pathophysiology may be orthostatic dysfunction or psychogenic vertigo. In the c-JSER, the diagnosis of definite MD does not consider the level of hearing. So, if the c-JSER had numerical audiological diagnostic criteria, all patients in this subgroup would have been excluded from the diagnostic criteria in the same way as the Bárány Society criteria. Therefore, the head-up tilt test, the Schellong test which is also a test for orthostatic dysregulation (26), dizziness handicap inventory, and other inner ear function tests (predictive of negative results) should be performed.

From the above, it can be concluded that in >80% of definite MD using the c-JSER, EH is the underlying pathology. If the patients diagnosed with definite MD claim cochlear symptoms during vertigo attacks but do not show any audiological abnormality or nystagmus, we must consider the possibility of orthostatic dysfunction or psychogenic vertigo. Ten percent of the patients with definite MD are likely to be diagnosed with VM in the future (25), so when the treatment for MD is not effective, we must think of the possibility of VM and continue to ask questions relating to the migraine.

### 4.2 Affected side, the side with EH, and the direction of nystagmus

The MD-affected side in each patient was determined using a flowchart (Figure 4). Of the 51 cases, the affected side in 50 (98%) cases was determined using a relatively simple method (i.e., interview and audiometry). Furthermore, the affected side was an exact match in all 31 cases where EH was present on one side. This suggests that the side with cochlear symptoms may have EH and that interview and audiometry information are important for determining the affected side of MD. On the other hand, the direction of nystagmus varied. Although many patients exhibited IN (74%, 32/43), as previously reported (27), some also exhibited PN (26%, 11/43) (Figure 6). One patient had EH on the affected side, but all six recordings showed PN from the onset of the attack. Thus, EH could be the primary cause of PN. We recorded three times following a single vertigo attack (at the time the symptom disappeared, one hour after the symptom disappeared, and the day after the attack). Comparing the direction at the onset of the attack, if the other three recordings showed the reverse direction, we concluded the reversal of nystagmus occurred. The reversal of nystagmus was observed in 19 patients (44%). This recording method had three intermittent periods so another reversal may have occurred. We speculated there might have been many more cases where the direction had reversed. The majority of the patterns of nystagmus were IN to PN change, but some patients showed only PN, and other patients showed PN at the onset of the vertigo attack and IN later, so it might be possible to show PN at the onset of the vertigo attack. We cannot deny the possibility that the recorded PN was not the onset nystagmus but the reversed one, or that the affected side and the unaffected side have been determined as reversed.

### 4.3 Limitations

This study has certain limitations. First, an inherent limitation regarding recording timing during vertigo attacks existed, as it may have taken several minutes for the patient to become aware of the vertigo attack and begin the recording. The time elapsed from the patient’s awareness of the onset of the vertigo attack to the start of the recording ranged from as short as one minute to several minutes. We believe that the time from the onset of the vertigo attack to the start of recording never exceeds 10 min, even in longer instances. During this time, the direction of the nystagmus could have been reversed. Thus, the PN recorded in the nystagmus videos 1(Figure 5) could have started with IN. Second, we limited our study to patients who experienced at least one vertigo attack every 2 weeks. This restriction was introduced because if the frequency of vertigo attacks is low, the PNR rental period must be extended. Third, the PNR was provided for only 2 weeks, with a maximum observation period of 4 weeks. The small number of recording devices did not allow us to observe a single patient over an extended period. To address the second and third limitations, it would be useful to utilize smartphones (28–30), which have become ubiquitous in recent years, to capture nystagmus videos immediately following the onset of vertigo attacks. Once the procedure is integrated into a smartphone application, anyone can easily obtain nystagmus videos, which could improve the quality of medical treatment for vertigo. This could be achieved by making the application widely available to patients with vertigo and making nystagmus recordings a routine practice immediately after the onset of vertigo attacks. Finally, the interval between recording nystagmus and undergoing CE-MRI presents an issue. This is because a prolonged interval might lead to observations of two vertigo attacks stemming from different causes. In our study, the median interval was 48 days (Table 2), hence, it is reasonable to assume that the underlying causes of the vertigo attack during nystagmus recording and at the time of CE-MRI were largely consistent. MRI is widely used by doctors in various departments other than neurotologists. CE-MRI needs special protocols and analysis, we cannot order frequently so the time lag with PNR recordings would occur.

### 4.4 Generalizability

Since the method for participant selection and determination of the suitability criteria is in accordance with Table 1, reproducibility is considered to be ensured. In addition, since the PNR videos were recorded in a quality that identified the direction of nystagmus in all patients, we believe that the determination of nystagmus characteristics can be reproduced under the same conditions as in the present study.

In this study, the rental period of PNR was relatively short, ranging from 2 to 4 weeks. Since the frequency of vertigo attacks differs from patient to patient, the number of videos obtained within a certain period of time is expected to vary widely from patient to patient. Patients with a low number of videos or those classified in subgroups other than CMDN may be required to rent PNR for a longer period, but this is difficult because of the scarcity of equipment. With regard to the interval between the acquisition of nystagmus videos and CE-MRI of the inner ear, there was a variation from patient to patient, with the requirement that it be within one year. Ideally, the interval between the two should be as short as possible to ensure reproducibility, but this is difficult because the timing at which nystagmus videos can be acquired is unpredictable.

## 5 Conclusion

Obtaining vertigo attack recordings of patients with MD is challenging because MD vertigo attacks usually occur outside the hospital. We developed and used a PNR to collect nystagmus videos of vertigo attacks and stratified patients based on the nystagmus and CE-MRI findings. Thus, the cases were stratified into four subgroups: (i) a CMDN subgroup whose pathogenesis is presumed to be true MD, (ii) a subgroup with nystagmus but without EH, presumed to be early MD or VM overlap, (iii) a subgroup with EH but without nystagmus, where they should also belong to CMDN and (iv) a subgroup that has neither nystagmus nor EH and is presumed to be caused by something other than the inner ear, such as orthostatic dysregulation or psychogenic vertigo. Considering the affected side, the side with cochlear symptoms corresponded to the side with EH, and the direction of nystagmus immediately after the onset of the vertigo attack was mostly IN, but some showed PN. Furthermore, about half of the nystagmus was subsequently reversed. Therefore, directly determining the MD’s affected side based on the nystagmus’s direction is challenging.

In the future, devices such as smartphones can be used to record nystagmus, and the methodology utilized in this study can be applied to diagnose MD. Further video data accumulation and discussion of the treatment efficacy decisions for stratified patients can enable tailoring MD treatment according to the pathogenesis of each case.

## Data availability statement

The raw data supporting the conclusions of this article will be made available by the authors, without undue reservation.

## Ethics statement

The studies involving humans were approved by the Ethics Committee of the Osaka University Hospital. The studies were conducted in accordance with the local legislation and institutional requirements. The participants provided their written informed consent to participate in this study.

## Author contributions

YU: Data curation, Formal analysis, Writing – original draft. TI: Data curation, Formal analysis, Funding acquisition, Project administration, Resources, Writing – review & editing. KH-S: Data curation, Formal analysis, Resources, Writing – review & editing. YuO: Writing – review & editing. TS: Writing – review & editing. TK: Writing – review & editing. YoO: Writing – review & editing. HI: Conceptualization, Funding acquisition, Methodology, Supervision, Writing – review & editing.

## Glossary

**Table:** 

CE-MRI	contrast-enhanced magnetic resonance imaging
c-JSER	the Japan Society for Equilibrium Research criteria
CMDN	certain Menière’s disease with nystagmus
EH	endolymphatic hydrops
GVEMP	glycerol vestibular evoked myogenic potential
IN	irritative nystagmus
MD	Menière’s disease
PN	paralytic nystagmus
PNR	portable nystagmus recorder
VM	vestibular migraine

## References

[1] Sajjadi H Paparella MM. Meniere’s disease. Lancet. (2008) 372:406–14. doi: 10.1016/s0140-6736(08)61161-7 18675691

[2] Mancini F Catalani M Carru M Monti B. History of Meniere’s disease and its clinical presentation. Otolaryngol Clin N Am. (2002) 35:565–80. doi: 10.1016/s0030-6665(02)00017-8 12486840

[3] Shojaku H Watanabe Y Fujisaka M Tsubota M Kobayashi K Yasumura S. Epidemiologic characteristics of definite Ménière’s disease in Japan. A long-term survey of Toyama and Niigata prefectures. ORL J Otorhinolaryngol Relat Spec. (2005) 67:305–9. doi: 10.1159/000089413, PMID: 16374065

[4] Kitahara T Okamoto H Fukushima M Sakagami M Ito T Yamashita A. A two-year randomized trial of interventions to decrease stress hormone vasopressin production in patients with Meniere’s disease-a pilot study. PLoS One. (2016) 11:e0158309. doi: 10.1371/journal.pone.0158309, PMID: 27362705 PMC4928871

[5] Paparella MM Morizono T Matsunaga T. Kyoshiro Yamakawa, MD, and temporal bone histopathology of Meniere’s patient reported in 1938. Commemoration of the centennial of his birth. Arch Otolaryngol Head Neck Surg. (1992) 118:660–2. doi: 10.1001/archotol.1992.01880060110023, PMID: 1637546

[6] Iwasaki S Shojaku H Murofushi T Seo T Kitahara T Origasa H. Diagnostic and therapeutic strategies for Menière’s disease of the Japan Society for Equilibrium Research. Auris Nasus Larynx. (2021) 48:15–22. doi: 10.1016/j.anl.2020.10.009, PMID: 33131962

[7] Naganawa S Yamazaki M Kawai H Bokura K Sone M Nakashima T. Visualization of endolymphatic hydrops in Ménière’s disease after single-dose intravenous gadolinium-based contrast medium: timing of optimal enhancement. Magn Reson Med Sci. (2012) 11:43–51. doi: 10.2463/mrms.11.43, PMID: 22450386

[8] Webster KE Galbraith K Harrington-Benton NA Judd O Kaski D Maarsingh OR. Systemic pharmacological interventions for Ménière’s disease. Cochrane Database Syst Rev. (2023) 2023:CD015171. doi: 10.1002/14651858.CD015171.pub2, PMID: 36827524 PMC9948543

[9] Webster KE Lee A Galbraith K Harrington-Benton NA Judd O Kaski D. Intratympanic corticosteroids for Ménière’s disease. Cochrane Database Syst Rev. (2023) 2023:CD015245. doi: 10.1002/14651858.CD015245.pub2, PMID: 36847608 PMC9969957

[10] Webster KE Galbraith K Lee A Harrington-Benton NA Judd O Kaski D. Intratympanic gentamicin for Ménière’s disease. Cochrane Database Syst Rev. (2023) 2023:CD015246. doi: 10.1002/14651858.CD015246.pub2, PMID: 36847592 PMC9969977

[11] Pullens B Verschuur HP van Benthem PP. Surgery for Ménière’s disease. Cochrane Database Syst Rev. (2013) 2013:CD005395. doi: 10.1002/14651858.CD005395.pub3, PMID: 23450562 PMC7389445

[12] Chen BS Roberts DS Lekovic GP. Vestibular neurectomy for intractable vertigo: case series and evaluation of role of endoscopic assistance in retrolabyrinthine craniotomy. J Neurol Surg B Skull Base. (2019) 80:357–63. doi: 10.1055/s-0038-1670685, PMID: 31328081 PMC6639113

[13] Antonio SM Friedman R. Ménière’s Disease In: Jackler RK Brackmann DE, editors. Neurotology. 2nd ed: Mosby (2005). 621–38. doi: 10.1016/B978-0-323-01830-2.50045-6

[14] Leng Y Fan W Liu Y Xia K Zhou R Liu J. Comparison between audio-vestibular findings and contrast-enhanced MRI of inner ear in patients with unilateral Ménière’s disease. Front Neurosci. (2023) 17:1128942. doi: 10.3389/fnins.2023.1128942, PMID: 36992853 PMC10040662

[15] Melki SJ Li Y Semaan MT Zheng QY Megerian CA Alagramam KN. A mouse model validates the utility of electrocochleography in verifying endolymphatic hydrops. J Assoc Res Otolaryngol. (2014) 15:413–21. doi: 10.1007/s10162-014-0445-0, PMID: 24509791 PMC4010592

[16] Nagarajan P Thangaraj MS. Effect of glycerol administration on ECochG and VEMP findings in individual with Meniere’s disease. Indian J Otolaryngol Head Neck Surg. (2022) 74:4110–6. doi: 10.1007/s12070-021-02856-7, PMID: 36742890 PMC9895202

[17] Ferraro JA Durrant JD. Electrocochleography in the evaluation of patients with Ménière’s disease/endolymphatic hydrops. J Am Acad Audiol. (2006) 17:045–68. doi: 10.3766/jaaa.17.1.6 16640060

[18] Ban JH Lee JK Jin SM Lee KC. Glycerol pure tone audiometry and glycerol vestibular evoked myogenic potential: representing specific status of endolymphatic hydrops in the inner ear. Eur Arch Otorhinolaryngol. (2007) 264:1275–81. doi: 10.1007/s00405-007-0370-5, PMID: 17598122

[19] Nakashima T Naganawa S Teranishi M Tagaya M Nakata S Sone M. Endolymphatic hydrops revealed by intravenous gadolinium injection in patients with Menière’s disease. Acta Otolaryngol. (2010) 130:338–43. doi: 10.1080/00016480903143986, PMID: 19685358

[20] Imai T Uno A Kitahara T Okumura T Horii A Ohta Y. Evaluation of endolymphatic hydrops using 3-T MRI after intravenous gadolinium injection. Eur Arch Otorhinolaryngol. (2017) 274:4103–11. doi: 10.1007/s00405-017-4739-9, PMID: 28948373

[21] Gürkov R Pyykö I Zou J Kentala E. What is Menière’s disease? A contemporary re-evaluation of endolymphatic hydrops. J Neurol. (2016) 263:71–81. doi: 10.1007/s00415-015-7930-1, PMID: 27083887 PMC4833790

[22] Conte G Lo Russo FM Calloni SF Sina C Barozzi S Di Berardino F. MR imaging of endolymphatic hydrops in Ménière’s disease: not all that glitters is gold. Acta Otorhinolaryngol Ital. (2018) 38:369–76. doi: 10.14639/0392-100x-1986, PMID: 30197428 PMC6146579

[23] Mammarella F Zelli M Varakliotis T Eibenstein A Pianura CM Bellocchi G. Is electrocochleography still helpful in early diagnosis of Meniere disease? J Audiol Otol. (2017) 21:72–6. doi: 10.7874/jao.2017.21.2.72, PMID: 28704892 PMC5516694

[24] Nakada T Yoshida T Suga K Kato M Otake H Kato K. Endolymphatic space size in patients with vestibular migraine and Meniere’s disease. J Neurol. (2014) 261:2079–84. doi: 10.1007/s00415-014-7458-9, PMID: 25099513

[25] Lempert T Olesen J Furman J Waterston J Seemungal B Carey J. Vestibular migraine: Diagnostic criteria. J Vestib Res. (2012) 22:167–72. doi: 10.3233/ves-2012-0453 23142830

[26] Fanciulli A Campese N Wenning GK. The Schellong test: detecting orthostatic blood pressure and heart rate changes in German-speaking countries. Clin Auton Res. (2019) 29:363–6. doi: 10.1007/s10286-019-00619-7, PMID: 31273549

[27] Meissner R. Behavior of the nystagmus in Menière’s attack. Arch Otorhinolaryngol. (1981) 233:173–7. doi: 10.1007/BF00453641 6976167

[28] Kıroğlu M Dağkıran M. The role of mobile phone camera recordings in the diagnosis of Meniere's disease and pathophysiological implications. J Int Adv Otol. (2020) 16:18–23. doi: 10.5152/iao.2019.6605, PMID: 32066548 PMC7224432

[29] Young AS Lechner C Bradshaw AP MacDougall HG Black DA Halmagyi GM. Capturing acute vertigo: A vestibular event monitor. Neurology. (2019) 92:e2743–53. doi: 10.1212/wnl.0000000000007644, PMID: 31092626

[30] Cambridge Digital Health. DizzyCam (2022). Available at: https://www.cambridgedigitalhealth.co.uk/dizzycam-app-page

